# Functional outcomes and quality of life following surgical treatment of aneurysmal bone cysts of the pelvis in children

**DOI:** 10.1007/s11832-014-0588-x

**Published:** 2014-05-11

**Authors:** Eduardo N. Novais, Amy K. Zimmerman, Laura W. Lewallen, Peter S. Rose, Franklin H. Sim, Amy L. McIntosh

**Affiliations:** Mayo Clinic, 200 First St. SW, Rochester, MN 55905 USA

**Keywords:** Aneurismal bone cyst, Pelvis, Children, Curettage, Bone grafting, Quality of life, Functional outcome

## Abstract

**Background:**

Aneurysmal bone cysts (ABCs) are a benign aggressive tumor that occurs rarely in the pelvis in the pediatric population. Pelvic ABCs may involve the triradiate cartilage and/or the acetabulum, which increases the technical difficulty of surgical treatment and has potential implications on the growth and development of the hip joint. This study examines the clinical presentation, rate of surgical complications, and recurrence rate, as well as, long-term clinical and functional outcomes of children with pelvic ABCs treated at a single institution by a single treatment modality.

**Methods:**

Between 1988 and 2008, 142 children with histologically confirmed ABCs were treated at our institution. Seventeen (12 %) tumors were located in the pelvis. A total of 13 pelvic ABCs (5 ilium-periacetabular, 4 pubic, 3 ilium-iliac wing, and 1 ischium) were included in this study. There were eight male and five female patients with a mean age of 12.9 years (range 4.1–17.5 years) at the time of surgery. The Toronto Extremity Salvage Score (TESS), the Musculoskeletal Tumor Society 1993 (MSTS’93) score, and the Short Form Health Survey Sf-36 were obtained at a minimum 5-year follow-up in all patients (mean follow-up 11.5 years, range 5.5–19.8 years). The mean age at follow-up was 24.3 years (range 14.6–32.6 years).

**Results:**

All patients were treated surgically with intralesional curettage extended with a high-speed burr and bone grafting. Eight patients received adjunctive therapy with phenol. Five patients had preoperative selective arterial embolization. Of the 13 patients, 1 had a local recurrence diagnosed at 6 months after surgery. The only complication in the cohort was a superficial wound infection. At the latest follow-up, all patients were free of disease. The mean TESS score was 95 and the mean MSTS’93 score was 93 %. The mean self-rated general health score, according to the SF-36 was 87 % of total points possible.

**Conclusions:**

Extended curettage and bone grafting of pelvic ABCs in the pediatric population can yield high clinical and functional scores at an average of 11 years follow-up with a low rate of complications and recurrence.

**Level of evidence:**

IV, case series.

## Introduction

Aneurysmal bone cysts (ABCs) are benign, highly vascular osseous lesions of unknown origin. Primary ABCs are rare, representing 1.4 % of all primary bone tumors [1]. ABCs are more often encountered in children and adolescents, with more than 70 % of the cases occurring during the first two decades of life [2, 3]. The most common location is the metaphysis of the long bones, specifically the distal femur and proximal tibia [2] and the spine [4, 5]. Pelvic location is rare. Only 4–9 % of all ABCs in pediatric patients are located in the pelvis [4, 6, 7].

Aneurysmal bone cysts are categorized as locally aggressive lesions that rarely undergo spontaneous healing [1, 8]. Potential treatment modalities include direct injection of the cyst with a fibrosing agent, embolization, resection and intralesional curettage with or without local adjuvant therapies [2, 4, 7–13]. With intralesional curettage, lower recurrence rates are reported with the addition of adjuvant therapy. These adjuvant therapies include: the use of a high-speed burr, application of phenol or liquid nitrogen, and argon beam coagulation [3, 4, 10, 11, 14]. The void in the bone can be filled with bone graft (allogenic or autogenic), bone cement, or calcium phosphate bone substitutes. The reported recurrence rate of ABCs located in the pelvis after surgical treatment is around 14 % [8, 15, 16]. Treatment of pelvic ABCs in children may be challenging because of the relative inaccessibility of the lesions, associated intraoperative bleeding, and the proximity of the lesions to neurovascular structures [16]. Furthermore, the lesion or the surgical treatment may disrupt the integrity of the triradiate cartilage or the acetabular cartilage and impact the development of the hip joint, with long-term consequences.

Most studies reporting on outcomes after surgical treatment of ABC of the pelvis focus on the short-term symptomatic relief, postsurgical complication, and recurrence rate [8, 15, 16]. Limited evidence in the literature on the long-term clinical and functional outcomes of surgical treatment of pediatric pelvic ABC during exists. The purposes of this study were (1) to report the clinical presentation, surgical treatment, rate of complication and recurrence; and (2) to describe the intermediate to long-term functional outcomes assessed by the Toronto Extremity Salvage Score (TESS) [17, 18] and the Musculoskeletal Tumor Society 1993 score system (MSTS’93) [19] and quality of life assessed by the Medical Outcomes Study Short Form Health Survey (SF-36) [20] in pediatric patients with ABC of the pelvis.

## Materials and methods

### Study sample

After institutional review board approval, we performed a query of the surgical tumor registry at our institution to identify patients who underwent treatment for a histologically confirmed ABC from 1988 to 2008. Inclusion criteria were age ≤18 years old and tumors located in the pelvis, excluding the sacrum. The registry query yielded 142 pediatric patients with surgically treated ABCs within the 20-year period. Seventeen (12 %) ABCs were located in the pelvis. We attempted to contact all patients using information from the medical records; four patients could not be located. The remaining 13 patients agreed to participate and completed a clinical questionnaire. A total of 13 pelvic ABCs were included in this study. There were eight male and five female patients with a mean age of 12.9 years (range 4.1–17.5 years) at the time of surgery. The minimum follow-up was 5.5 years (mean 11.5 years, range 5.5–19.8 years). The mean age at follow-up was 24.3 years (range 14.6–32.6 years).

**Fig. 1:**
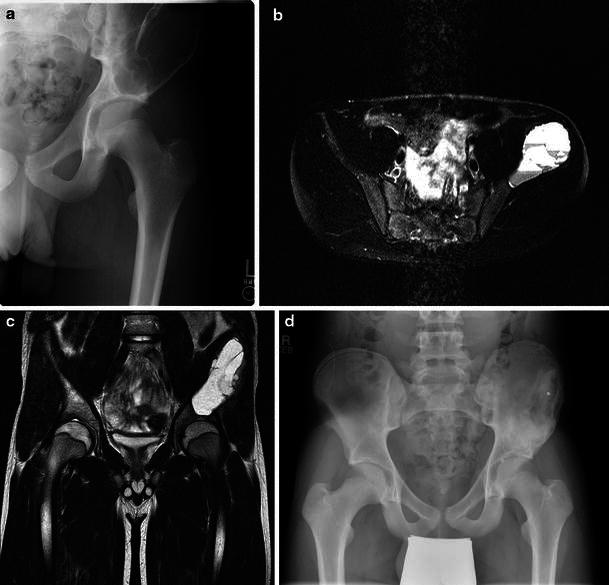
Aneurysmal bone cyst (ABC) of the left iliac wing adjacent to the acetabulum in a 14-year-old-male that presented with worsening left hip pain in the past 8 months. **a** Anteroposterior radiograph reveals a very large expansile, osteolytic lesion of the left ilium extending into the superior acetabulum. **b** Axial T2-weighted magnetic resonance imaging (MRI) reveals multiple fluid levels characteristic of an aneurismal bone cyst. **c** Coronal T2-weighted MRI demonstrating extension into the supra-acetabular region with no involvement of the joint. **d** Anteroposterior pelvic radiograph 2 years postoperative reveals no signs of local recurrence and complete bone healing of the entire cystic area. The patient at most recent follow-up was very active and had returned to high school baseball, wrestling, and football without limitation

The medical clinical records were reviewed to record type and length of symptoms at initial presentation, age at surgery, surgical technique, pathology reports, complications and recurrence of the lesion. Preoperative and postoperative plain radiographs obtained at a minimum of 1 year after treatments were available for all patients. The lesions were staged according to the system described by Enneking [21]: Stage-1 (latent) lesions are well marginated by cortical reactive bone; Stage-2 (active) lesions expand by deformation with bulging of the overlying cortical bone; and Stage-3 (aggressive) lesions invade by destroying the restraining bone and permeating with rapid extension into adjacent tissues. Lesion size was measured on radiographs using the widest dimension as reference, and was dichotomized in ≤5 or >5 cm according to the criteria of Papagelopoulos et al. [16]. Preoperative computed tomography (CT) and magnetic resonance imaging (MRI) were reviewed when available. The most recent anteroposterior and lateral pelvic radiographs obtained at a minimum of 1 year after treatment were assessed to evaluate healing (Fig. 1). A lesion was considered radiographically healed when it was completely filled with bone [8].

Functional outcomes were measured using the TESS [17, 18] and the MSTS’93 score system [19]. The TESS is a tool developed to evaluate physical function after surgical treatment of musculoskeletal tumors of the extremities that emphasizes the patient’s perspective on their physical abilities over time. Assessment includes limitations on activities of daily living, and work/school, sports/recreation, and mobility [17, 18]. The MSTS’93 was designed as a standardized clinical evaluation of musculoskeletal tumor reconstructive surgeries that addresses physical pain, occupational and recreational functional limitations, emotional acceptance of treatment outcomes, and extremity-specific physical function. Each item is rated on a 0–5 scale corresponding to poor, fair, good, or excellent for a maximum total score of 35. Results may be reported as a percentage of total possible points. Higher scores reflect greater function [19]. Quality of life and general health status were evaluated using the Medical Outcomes Study Short Form Health Survey (SF-36), a generic measure designed to detect medically and socially relevant change over time [20]. Patients rate individual items on a Likert-type scale; cumulative results are represented on a scale of 0–100 with higher scores reflecting greater health and well-being.

## Results

### Clinical and imaging presentation

The most common presenting symptom was hip pain (Table 1). The average duration of symptoms prior to diagnosis was 4.8 months (range 0–12 months). One patient with a periacetabular iliac ABC presented with anterior pelvic pain irradiating to the testicles and had been previously evaluated and treated by pediatric urology without symptom improvement. Most lesions were categorized as latent and appeared expansile and lytic on plain radiographs. Computerized tomography was obtained for 11 patients and revealed the characteristic expansile nature of the lesions. MRI was obtained in six of the cases; all of which demonstrated characteristic multiple fluid levels and internal septations. The tumor involved the ilium in eight patients. Three of these lesions were restricted to the iliac wing while in five patients the lesion was adjacent to the acetabulum. Two periacetabular lesions were found to be intra-articular causing subluxation of the hip in one patient. Four lesions were found in the pubis, one of which expanded from the pubic symphysis toward the anterior column of the acetabulum. One lesion was restricted to the ischium and three lesions involved the triradiate cartilage.

**Table 1:** Demographics, clinical presentation and radiographic characteristics of 13 children with pelvic aneurysmal bone cyst (ABC). *MRI* Magnetic resonance imaging

Case	Sex	Age (years)	Presentation	Presenting symptoms	Symptoms duration (months)	Lesion location	Radiographic findings	Computed tomography	MRI
1	F	10.5	Primary	Hip pain, inability to bear weight	7	Ilium Peri-acetabular	Expansile lytic lesion with hip subluxation	Large expansile osteolytic lesion with a soft tissue component	None
2	M	6.1	Primary	Hip pain	12	Pubis	Expansile lytic lesion	Large expansile osteolytic lesion	None
3	M	17.5	Recurrent	Hip pain	6	Ilium Peri-acetabular	Expansile lytic lesion	Large expansile osteolytic recurrent lesion	None
4	F	15.1	Primary	Accidental finding after MV accident	–	Pubis	Expansile lytic lesion with pathologic fracture	None	None
5	M	16.3	Primary	Hip pain	2	Ischium	Non-expansile well defined lesion	Well-circumscribed non-expansile osteolytic lesion with mild cortical disruption	Well defined lesion with heterogeneous signal, fluid–fluid levels
6	M	13.9	Primary	Hip pain, pathologic fracture	0.5	Pubis	Expansile lytic lesion with pathologic fracture	Expansile osteolytic lesion and pathologic fracture	Expansile lesion with internal septations and fluid–fluid levels
7	F	15.8	Primary	Accidental finding during evaluation for nephrolithiasis	–	Ilium	Expansile lytic septate lesion	Expansile osteolytic, some fibrous septae within	Fluid filled expansile lesion with internal septations; fluid–fluid levels
8	M	4.1	Primary	Limp	6	Ilium	Expansile lytic lesion with cortical destruction	Large expansile osteolytic lesion with a thin expanded cortex with thick periosteal reaction	Fluid filled expansile lesion with internal septation and fluid–fluid levels with a soft tissue mass
9	M	12.6	Primary	Hip pain, pathologic fracture	0	Ilium Peri-acetabular	Expansile lytic septate lesion	Expansile osteolytic lesion	None
10	F	9.9	Recurrent	Hip pain	16	Ilium Peri-acetabular	Expansile lytic lesion with cortical destruction	Large expansile osteolytic lesion	Fluid filled expansile lesion with internal septation and fluid–fluid levels
11	M	17.5	Primary	Hip pain	6	Ilium—iliac wing	Expansile lytic lesion	Large expansile osteolytic lesion	None
12	M	13.6	Primary	Pelvic pain and limp	1	Ilium, periacetabular	Expansile lytic lesion	Large expansile osteolytic lesion with multiple internal septations	None
13	F	14.7	Primary	Groin pain	5	Pubis	Expansile lytic lesion	None	Fluid filled expansile lesion with internal septation and fluid–fluid levels

### Treatment

Eleven patients were treated for a primary lesion, two patients (patients 3 and 10) presented with a recurrent ABC treated previously elsewhere. Five patients had a diagnostic biopsy before surgery. In the remaining eight patients, frozen section histologic confirmation was obtained during surgery. All patients underwent curettage of the lesion through a large cortical window. Curettage was extended to further expose healthy bone beyond the reactive zone around the tumor with the use of a high-speed burr. In the majority of patients (8/13) phenol adjuvant therapy was used. A mixture of bone allograft and local autograft was used to fill the lesional cavity. Five patients underwent preoperative selective arterial embolization (SAE) after the vascularity and size of the feeding vessels were identified by arterial angiography. One patient with a very large peri-acetabular lesion and hip subluxation was immobilized in a spica cast after surgery.

### Clinical and functional outcomes

At the time of final follow-up, all patients were free of disease (Table 2). The lesion was found to be completely healed when the previous lesion cavity was completely filled with new bone with advanced cortical thickening in the periphery. One patient (patient 3) developed a local recurrence 6 months after treatment of a recurrent iliac tumor previously treated elsewhere. This patient underwent repeated extended curettage, high-speed burring, phenol and bone grafting. The lesion healed uneventfully and at 11.6 years after surgery he remains asymptomatic with high functional and quality of life scores. The only surgical complication in this series was a superficial wound infection (patient 10) that was treated with oral antibiotics and dressing changes.

**Table 2:** Treatment, functional outcomes and quality of life of 13 pediatric patients with pelvic ABC

Pt	Stage [11]	Treatment	SAE	Recurrence (months) treatment	Length FUP (years)	Age at FUP (years)	MSTS’93	TESS	SF-36 aggregate score
1	3	EXBG	Yes	None	17.3	27.8	97	100	92
2	2	EXBG	No	None	19.8	25.9	97	100	94
3	3	EXBG + phenol	Yes	Yes (6) EXBG	11.6	29.1	80	100	89
4	3	EXBG + phenol	Yes	None	16.1	31.2	60	85	62
5	2	EXBG	No	None	6	22.3	100	100	87
6	2	EXBG	No	None	10.5	24.4	100	100	94
7	2	EXBG + phenol	No	None	6.5	22.3	100	90	90
8	3	EXBG + phenol	Yes	None	10.5	14.6	100	100	92
9	2	EXBG + phenol	No	None	10.8	23.4	100	100	95
10	3	EXBG + phenol	Yes	None	10.8	20.7	100	95	91
11	2	EXBG + phenol	No	None	5.5	23.0	100	99	88
12	2	EXBG + phenol	No	None	5.7	19.3	70	65	67
13	2	EXBG	No	None	17.9	32.6	100	100	95

*PT* Patient, *EXBG* curettage extended by the use of a high-speed burr + bone grafting, *RES* marginal resection, *FUP* follow up, *SAE* preoperative selective arterial embolization

The mean TESS score was 94.9 (range 65–100, SD 10.2). Eleven patients reported no restrictions in their overall ability to perform activities of daily living; one patient experienced ongoing limitations from postsurgical pain, stiffness, fatigue, weakness, and ROM. Two patients reported difficulty with functional movements requiring significant hip flexion such as walking up stairs, bending, and picking items up off the floor. The mean MSTS’93 score was 92.6 % (range 60–100 %, SD 13.6 %). Nine patients experienced no pain; 2/13 experience non-disabling pain; 1/13 experienced modest pain controllable with non-narcotic analgesics; and 1/13 experienced moderate or intermittently disabling pain. Twelve patients scored the highest rating (‘enthusiastic’) for emotional acceptance of surgical outcomes; only one patient was ‘satisfied’. All 13 patients would recommend their surgical treatment to others with the same problem.

The mean self-rated general health score, according to the SF-36, was 87.4 % (range 62–95 %, SD 10.5 %). The mean physical function sub-scale score was 92 % (range 55–100 %, SD 16.8 %); 11 (84.6 %) patients felt that their physical health did not limit them in any way in performing common activities such as running, climbing stairs, walking, or self-care. The average social roles and physical health sub-scale score was 90 % (range 25–100 %, SD 24 %); 11 (84.6 %) patients did not feel that their physical health interfered in any way with their ability to fully participate in work or recreational pursuits. The average bodily pain sub-scale score was 89 % (range 35–100 %, SD 19.2 %); eight (61.5 %) patients had not experienced any bodily pain during the previous 4 weeks and had not experienced any interference with normal work inside or outside the home secondary to bodily pain.

## Discussion

Aneurysmal bone cysts are benign, although often destructive, osteolytic lesions that can be difficult to treat when located in the pelvis. The presence of open growth plates in children has been associated with a high recurrence rate after treatment of ABCs [4, 9, 22]. Treatment of an aneurismal bone cyst in the pediatric pelvis is a therapeutic challenge because of the presence of the triradiate cartilage, the destructive nature of the lesion that can involve the acetabulum and the difficult anatomic access. Few previous studies on pelvic ABCs reported on short-term clinical results focusing primarily on treatment complications and tumor recurrence [8, 15, 16]. In this retrospective study, 13 pediatric patients were assessed to determine clinical presentation, surgical complications, local recurrence, functional outcomes and quality of life following an average of 11.5 years after surgical treatment of an ABC of the pelvis.

Aneurysmal bone cysts involving the pelvis are rare in children. The reported rate of ABCs involving the pelvis in children varies and range from 4 % to 9 % [4, 6, 7]. In this study, we found a slighter higher incidence—12 % of 142 pediatric ABCs were located in the pelvis. Cottalorda et al. [15] reported a series of 15 children with pelvic ABCs and reviewed 31 additional cases available in the French and English literature. The most common presenting symptom was hip pain often followed by a limp [2, 8, 15, 16], which was also observed in this study. Pelvic ABCs can present a diagnostic challenge because the close relation with pelvic organs may lead to non-specific pelvic or abdominal symptoms [23]. In this series, one patient presented with anterior pelvic pain irradiating to the testicles and had been evaluated and treated previously by pediatric urology without symptom improvement. The deep location may allow the tumor to achieve considerable dimensions before it becomes symptomatic. In our series the average duration of symptoms was 5 months, which was very similar to the 4-month delay in diagnosis previously reported [15].

Previous reports on all sites of ABCs showed that recurrence is directed related to the surgical technique with a higher rate of local control when adjuvant therapy [3, 4, 10] such as the use of a high-speed burr, phenol, cement or liquid nitrogen is applied compared to curettage alone [14]. Although this study spans a 20-year period, the treatment described here was very consistent in most patients. All patients underwent curettage through a wide cortical window that was extended to further expose healthy bone beyond the reactive zone around the tumor with the use of a high-speed burr. Adjunctive therapy with chemical cauterization with phenol was performed in eight patients. In all cases the tumor cavity was filled with allograft mixed with local autograft. SAE was performed 1 day before surgery in all Stage-3 (aggressive) lesions with cortical bone destruction and extension into adjacent soft tissue mass. With this treatment strategy there was only one case (7.7 %) of local recurrence, which is the same rate as that reported by Capanna et al. [8] for surgically treated pelvic ABCs. Cottalorda et al. [15] reported a slightly higher rate (14 %) of local recurrence in pediatric pelvic ABCs. The local recurrence in our series was treated with repeated curettage with extension of the margins with a high-speed burr and chemical cauterization with phenol and bone grafting. We did not use radiation therapy because of the risk of radiation-induced sarcoma [24]. In addition, radiation therapy for the treatment of pelvic ABCs is associated with a high risk of complications including femoral head osteonecrosis, pelvic deformity and leg-length discrepancy [8].

Previous studies on the outcomes after treatment of ABCs of the pelvis focused on surgical complication and local recurrence with short-term results [8, 15, 16]. In a previous series from the author’s institution Papagelopoulos et al. [16] reported on 40 consecutive patients (treated between 1921 and 1996) with ABCs involving the pelvis and/or sacrum with average follow-up of 13 years. Although no functional outcome was used, all patients were disease-free and 70 % of them were reported to be asymptomatic. Four patients in the current series were included in that previous study. Capanna et al. [8] reported on 23 cases of pelvic ABCs reviewed after a mean of 7 years and report a high rate of complication and functional deterioration in patients who underwent radiation therapy. Cottalorda et al. [15] analyzed 15 pelvic ABCs in children and adolescents with an average follow-up of 50 months and showed that all patients had normal function without discomfort; no validated objective outcome measurement of functional performance was used, however. In the current series, we report a high average level of function assessed by the TESS score and the MSTS’93 score as well as a high mean quality of life score assessed by the SF-36 at mean of 11 years following surgical treatment of pediatric pelvic ABCs. Using the TESS, the MSTS’93 and the SF-36 (as described above), mean scores were 94.9, 92.6, and 87.4 %, respectively. Evaluating functional outcomes after surgical management of musculoskeletal tumors is an important way of gaining information on patients’ perceptions. Given the technically challenging nature of treating ABCs of the pelvis in patients who are still growing and developing, this additional information can help determine the success of intervention. Upon completion of the TESS, nearly 85 % of patients reported no restrictions in their activities of daily living, no difficulty completing tasks for work or school, and viewed themselves as “not at all disabled.” The SF-36 also showed that nearly 85 % of patients felt that their physical health was not limited in any way. Additionally, all of the sub-scale scores (physical function, average social roles and physical health, and bodily pain) for the SF-36 were between 89 % and 92 %. Approximately 70 % of patients who completed the MSTS reported no pain related to their pelvic ABC surgery. All patients would recommend surgical treatment to others with the same problem.

We acknowledge several limitations of the current study including a small sample size, although this is difficult to overcome since pelvic ABCs are relatively rare in children [4, 6, 7]. The small sample size prevents strong inference, as well as the development of statistical models and investigation of potential predictors of the clinical and functional outcomes. With the number of patients available, it was not possible to analyze the influence of specific ABCs features including involvement of the triradiate cartilage and/or acetabulum. Only 3/10 patients had involvement of the triradiate cartilage and 2/13 had intra-articular tumor. These patients did not present with lower functional or quality of live scores at the most recent follow up. However, further investigation is needed to assess whether involvement of the triradiate cartilage or the hip joint have a negative impact on the radiographic morphology of the hip on a long-term. One limitation of the TESS and MSTS’93 scores is that they were developed to assess the impact of reconstructive surgical intervention for treatment of sarcoma of the lower extremities. In addition, the outcomes scores could not be used to assess patient improvement, since there were no preoperative scores. We believe, however, that the scores represent self-reported patient quantitative data about the function and quality of life at most recent follow-up. Although the outcome measures have not been validated in pediatric patients, most patients were adults at the time of data collection. The mean age at follow up was 24.3 years (range 14.6–32.6 years). Due to the retrospective nature of this study, there was variability in treatment. Adjuvant phenol therapy at time of curettage and pre-operative SAE were not applied consistently in every patient. The senior authors liberally employed preoperative embolization with no specific criteria during the study period. With the numbers available, we cannot provide a robust statistical analysis of the outcome of tumors treated with or without phenol adjunctive therapy and with or without preoperative embolization. We currently use adjunctive therapy with either phenol or more recently argon beam coagulation [11] and preoperative embolization of ABCs located in the pelvis based on clinical experience, even though our numbers were too small to prove their benefit statistically.

Surgical treatment of pelvic ABCs in children should be planned based on location, size and aggressiveness of the lesion. Long-term satisfactory clinical and functional outcomes can be expected after surgical treatment of ABCs of the pelvis in children.
